# Ferroelectric Domain Studies of Patterned (001) BiFeO_3_ by Angle-Resolved Piezoresponse Force Microscopy

**DOI:** 10.1038/s41598-017-18482-9

**Published:** 2018-01-09

**Authors:** Bumsoo Kim, Frank P. Barrows, Yogesh Sharma, Ram S. Katiyar, Charudatta Phatak, Amanda K. Petford-Long, Seokwoo Jeon, Seungbum Hong

**Affiliations:** 1 Department of Materials Science and Engineering, KAIST, Daejeon, 305-701 Korea; 2 Materials Science Division, Argonne National Laboratory, Lemont, IL 60439 USA; 3 Department of Physics and Institute for Functional Nanomaterials, University of Puerto Rico, San Juan, PR-00936-8377 USA; 4 Applied Physics Program, Northwestern University, Evanston, IL 60208 USA; 5 Materials Science and Engineering Department, Northwestern University, Evanston, IL 60208 USA

## Abstract

We have studied the ferroelectric domains in (001) BiFeO_3_ (BFO) films patterned into mesas with various aspect ratios, using angle-resolved piezoresponse force microscope (AR-PFM), which can image the in-plane polarization component with an angular resolution of 30°. We observed not only stable polarization variants, but also meta-stable polarization variants, which can reduce the charge accumulated at domain boundaries. We considered the number of neighboring domains that are in contact, in order to analyze the complexity of the ferroelectric domain structure. Comparison of the ferroelectric domains from the patterned and unpatterned regions showed that the elastic relaxation induced by removal of the film surrounding the mesas led to a reduction of the average number of neighboring domains, indicative of a decrease in domain complexity. We also found that the rectangular BFO patterns with high aspect ratio had a simpler domain configuration and enhanced piezoelectric characteristics than square-shaped mesas. Manipulation of the ferroelectric domains by controlling the aspect ratio of the patterned BFO thin film mesas can be useful for nanoelectronic applications.

## Introduction

Ferroelectric oxide materials are of great interest because of a demand for next-generation memory devices, actuators, energy harvesters, transducers, and microelectromechanical (MEMS) systems applications^1^. Among various materials candidates, the lead zirconate titanate (Pb(Zr,Ti)O_3_, PZT) family is the most studied for applications in nonvolatile memories and piezoelectric actuators^2,3^. An increased piezoelectric response was reported for PZT nanostructures due to the removal of the clamping effect and the enhanced mobility of domain walls^4,5^. PZT, however, has a critical drawback because the toxicity of lead creates environmental and safety issues^6^. Recently, the lead-free ferroelectric BiFeO_3_ (BFO) has received great attention due to its superior thin-film ferroelectric properties and high ferroelectric Curie temperature (T_c_ = 850 °C in single crystals)^7–10^. In applying BFO to data storage technology, thin, nanostructured BFO films have been studied as a way of reducing operational voltage and increasing circuit density^11,12^. The effect of lateral size on nanostructured BFO was studied in films patterned using focused-ion-beam (FIB) milling with a sacrificial layer, and it was discovered that ferroelectricity was preserved in structures with sizes down to 250 nm^13–15^. Moreover, Johann *et al*. reported enhancement of the piezoelectric response in the BFO nanostructures^16^.

BFO thin films also have disadvantages such as high leakage currents, for which oxygen vacancies and the unusual local electronic transport behavior at ferroelectric domain walls are the main cause^17–19^. However, the formation of the domain boundaries in BFO and their effects on the piezoresponse at the nanoscale are not clear. Therefore, constructing accurate ferroelectric domain maps is important for understanding not only the position of the domain boundaries and domain configuration but also the mechanism of polarization switching and electrical properties. The most common approach is to use piezoresponse force microcopy (PFM), however, constructing three-dimensional PFM images of polarization domains has been a great challenge because of sliding of the tip and cantilever buckling and an asymmetric tip apex, in addition to sample issues such as surface morphology and surface adsorbates^20–22^. Previously, Park *et al*. introduced angle-resolved PFM (AR-PFM) which provides clearer information on the in-plane polarization directions than conventional PFM method^23–26^. More recently, this method was also extended to study charged domain boundaries and the effect of poling on domain structures^27^.

Here we present direct observation using AR-PFM of domain boundaries and intermediate polarization variants in epitaxially-grown BFO thin films patterned into mesas, and we discuss the correlation between the resulting domain configurations and piezoresponse switching behavior. To observe the effect of the elastic relaxation induced by FIB patterning, we imaged the ferroelectric domains of BFO thin films patterned with various aspect ratios. We introduced a new parameter called ‘number of neighboring domains’ (NND) to analyze the complexity of the ferroelectric domain map. We claim that the domain structures of patterned BFO mesa structures are less complicated than that of unpatterned BFO films based on the NND analysis. Moreover, high aspect ratio of BFO mesas have an ordered domain configuration and enhanced piezoelectric characteristics.

## Results and Discussion

**Figure 1:**
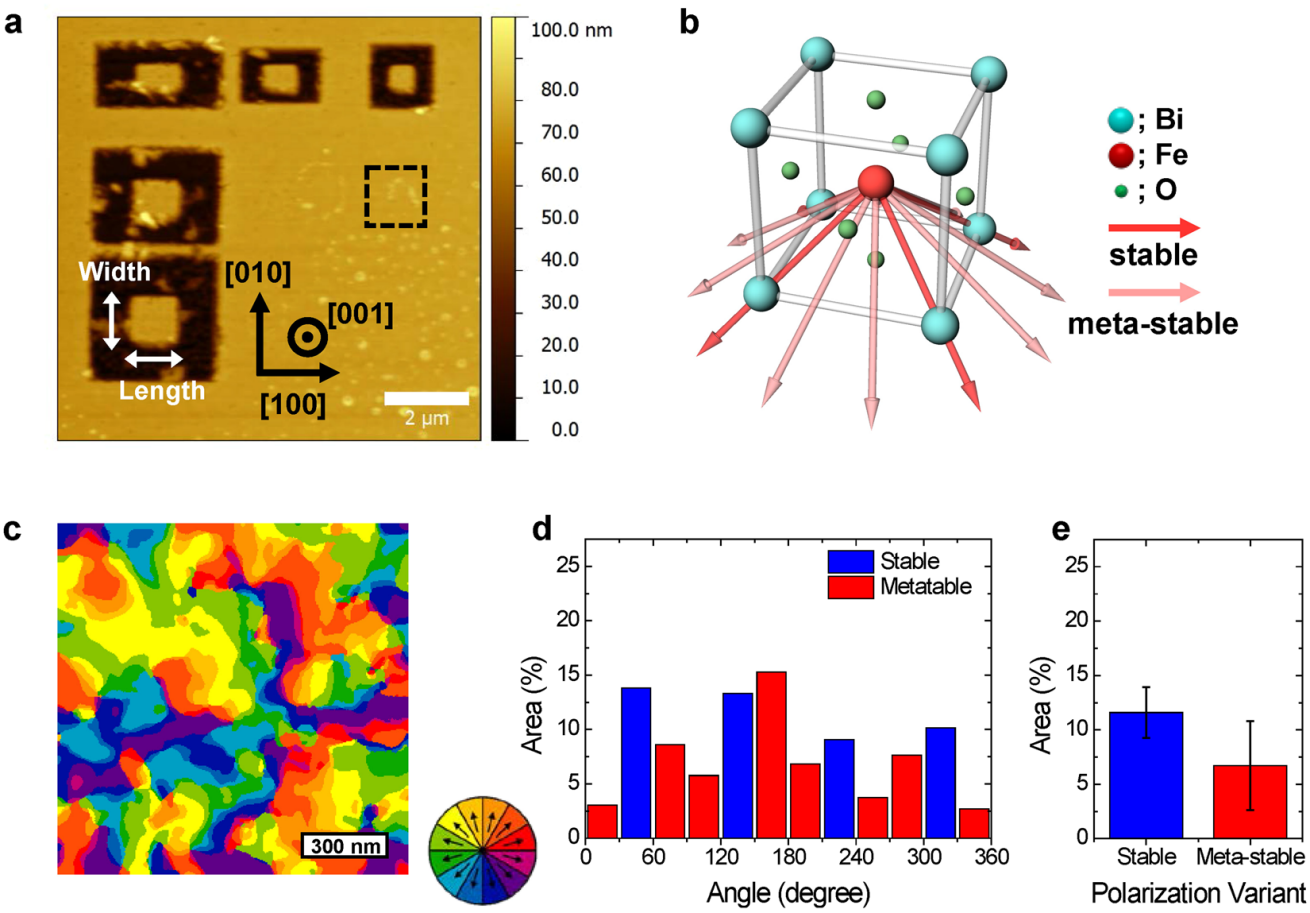
(**a**) Patterned mesas are separated from the continuous film by lithography, as shown in the AFM topography image. (**b**) Schematic drawing of the atomic structure of BFO with angle-resolved polarization models. The Fe (red sphere) atom can be displaced towards twelve possible polarization orientations with respect to its centrosymmetric position. (**c**) AR-PFM domain map of a 1.2 × 1.2 μm^2^ area of unpatterned BFO film, corresponding to the black dashed area in (**a**). (**d**) The area distribution of each polarization variant according to angle relative to the [100] direction. (**e**) The average area of stable and meta-stable polarization variants.

AR-PFM images were obtained of an epitaxial (35 nm) BFO/(70 nm) SrRuO_3_ (SRO) thin film heterostructure grown on a (001)-oriented STO substrate. The BFO film was patterned into mesas using combined electron-beam lithography and focused ion-beam (FIB) patterning with a removable tungsten mask for protecting from Ga implantation and knock-on damage from the ion-beam tails^12^. Figure 1(a) shows the AFM topography image of the patterned BFO mesas and Fig. S1 shows vertical PFM (VPFM) phase and amplitude images of the same region. The VPFM amplitude and phase of the etched region converges to zero and contains a noise signal, respectively, indicating that the BFO surrounding the mesas is completely etched and the patterned BFO mesas are discontinuous from the film.

Various studies have reported that BFO polarization vectors can adopt one of eight thermodynamically-stable variants along the <111> crystallographic directions. By also considering the polarization components determined using VPFM (see phase images in Fig. S1(a)), we can determine that the BFO thin film studied here had a uniform downward polarization vector component, and thus the possible polarization variants for the BFO thin film are restricted to the four stable variants shown by the red arrows of Fig. 1(b). Therefore, a conventional ferroelectric domain map can be constructed by combining lateral PFM (LPFM) phase images with the cantilever direction aligned along [100] and [010].

However, as we increase the angular resolution from 90° to 30°, we can detect twelve polarization variants, including eight meta-stable polarization variants that do not lie along the <111> directions, as shown by the pink arrows in Fig. 1(b). Figure S2(a)–(f) show LPFM phase images of the BFO thin film obtained after rotating the sample by increments of 30°. As the cantilever direction changed from 0° to 30°, from 60° to 90°, and from 90° to 120°, the total area that is black (or grey) changed by less than 15% and the overall shape of the domains was maintained. On the other hand, when the cantilever angle changed from 30° to 60° and from 120° to 150°, the domain shape changed significantly and the total area for each polarization variant was changed by over 20%. In Fig. 1(c)–(e), the AR-PFM domain map and analysis of the polarization variants in a 1.2 × 1.2 μm^2^ area of unpatterned BFO film are shown: the average area of each stable polarization variant is 11.6% while the average area of the eight meta-stable polarization variants is 6.7%. Meta-stable polarization variants lead to lower charge distribution along the domain walls (see accompanying explanation in Fig. S3), and the AR-PFM domain map shows a more accurate representation of the domain orientations and walls than would be achievable using standard PFM.

**Figure 2:**
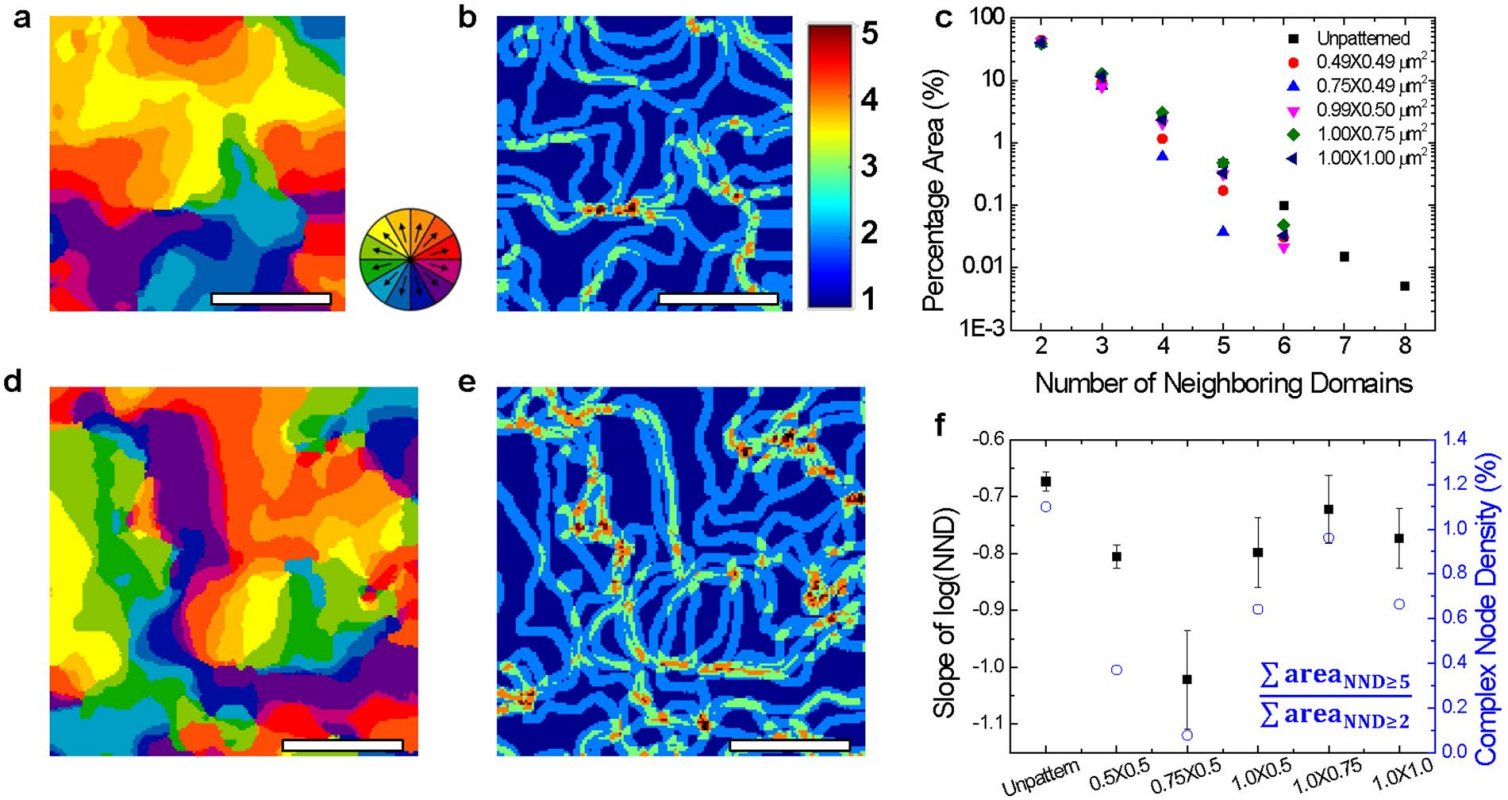
AR-PFM domain maps for (**a**) patterned and (**d**) unpatterned BFO thin film. NND maps for (**b**) 0.5 × 0.5 µm^2^ patterned mesa and (**e**) unpatterned BFO thin film. (**c**) Areal ratio of NND is plotted on a semi-logarithmic scale for D = 20 nm. The log-scaled area for the patterned BFO mesas decreases more rapidly than for the unpatterned BFO film. (**f**) Slope of lines shown in (**c**) and complex node density for patterned and unpatterned regions of BFO film. All scale bars are 200 nm.

In the case of Pb(Zr,Ti)O_3_ (PZT) nanostructures, the effective clamping stress changes when the length over thickness is below 100, and beyond ratios of 100:1 it remains constant^28^. In the system being studied here, the length over thickness of the BFO mesas ranges from 14 to 29, which is small enough so that clamping can be relieved sufficiently by removing the surrounding materials. In order to explore the effect of elastic relaxation on the ferroelectric domains, we conducted AR-PFM imaging of patterned and unpatterned regions of the BFO thin film as shown in Fig. 2(a),(d), respectively. To gain insights into the ferroelectric domains and their boundaries, we defined a parameter that we term NND, and which is the total number of neighboring domains within a circle of diameter *D* at a pixel position *i*:

1 $${{\rm{NND}}}_{i,D}={\rm{Number}}\,{\rm{of}}\,{\rm{neighboring}}\,{\rm{domains}}\,{\rm{within}}\,{\rm{diameter}}\,D\,{\rm{of}}\,{\rm{pixel}}\,i$$

We carried out this analysis at each pixel across our image, as a way of mapping the domain boundaries and of determining the complexity of the domain pattern. As the diameter of the circle increases NND_*i,D*_ will increase or remain constant as shown in Fig. S4. We then calculated the percentage area as a function of *x*, which is the percentage of all pixel areas for which the value of NND is *x*.

2 $${\rm{Percentage}}\,{\rm{area}}(x)=\frac{{\sum }_{i}NN{D}_{i,D}=x}{{\rm{Total}}\,{\rm{area}}}$$

The percentage area for the NND_*D*_ analysis showed similar trends for all values of *D* that we explored, decreasing exponentially as shown in Fig. S5 regardless of the diameter, within limits of 12 nm to 30 nm. We set *D* to 20 nm because the tip radius of curvature was <25 nm. Figure 2(b),(e) are the NND maps for the 0.5 × 0.5 μm^2^ patterned BFO mesa and the unpatterned region of the BFO film shown in Fig. 2(a),(d), respectively. In the NND map, a region having NND of two or more indicates a ferroelectric domain boundary, and a larger value of NND indicates a higher value of intersecting domains. Comparing the NND maps for the patterned and the unpatterned regions, we can see that the domain boundary density is similar (54% for the patterned BFO and 53% for the unpatterned BFO). However, the density of the complex regions where NND is greater than five, is three times higher in the unpatterned BFO film than for the patterned BFO film.

Figure 2(c) shows a log-linear plot of the percentage area corresponding to each NND. For NND = 2, the BFO mesas and the unpatterned BFO have a similar value of percentage area. However, as NND increases, the difference of percentage area between the patterned BFO mesas and the unpatterned BFO becomes clear, with the percentage area for the patterned mesas decreasing faster. Moreover, the maximum NND for the patterned mesas is 5 to 6 while the maximum NND for the unpatterned BFO is 8. These results indicate that the ferroelectric domain structure in the unpatterned BFO is more complex than in the patterned BFO mesas. We attribute this to the relaxation induced by removal of the film surrounding the BFO mesas. The equilibrium domain variants inside the mesa structure can tolerate a higher strain gradient, which enhances variations in the local strain and lattice rotation due to less constraint from strain confinement^12^.

In order to investigate the complexity of the domain boundaries, we compared the ratio of the percentage area with NND ≥ 5 or more to the percentage area with NND ≥ 2 by defining the complex node density:

3 $${\rm{Complex}}\,{\rm{node}}\,{\rm{density}}=\frac{{\sum }_{x=5}{\rm{percentage}}\,{\rm{area}}(x)}{{\sum }_{x=2}{\rm{percentage}}\,{\rm{area}}(x)}$$

Figure 2(f) shows the complex node density for the patterned and unpatterned regions of the BFO film. The unpatterned BFO film has a higher complex node density than the patterned mesas, again suggesting that the patterned mesas have a simpler ferroelectric domain structure than the unpatterned BFO film.

**Figure 3:**
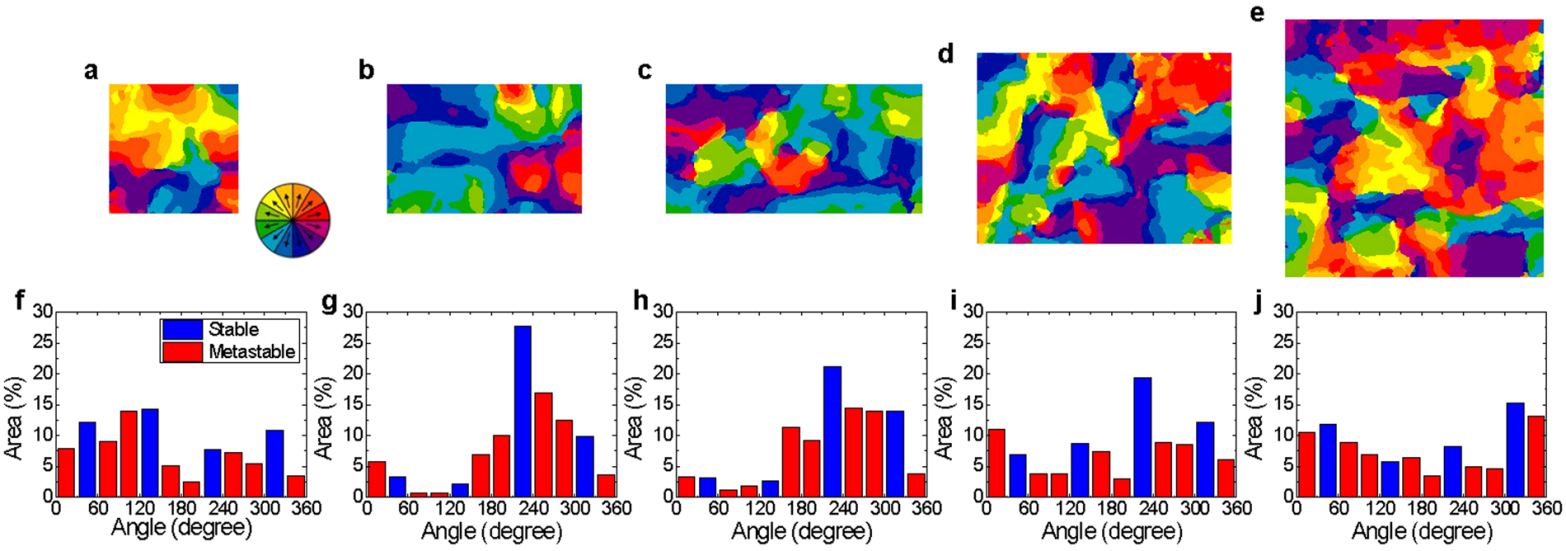
(**a**)–(**e**) In-plane ferroelectric domain maps for the patterned BFO thin film, constructed by AR-PFM. The mesa sizes are (**a**) 0.5 µm × 0.5 µm, (**b**) 0.75 µm × 0.5 µm, (**c**) 1.0 µm × 0.5 µm, (**d**) 1.0 µm × 0.75 µm, and (**e**) 1.0 µm × 1.0 µm. (**f**)–(**j**) Plots showing the distribution of area for each polarization variant according to angle relative to the [100] direction for the different patterned mesas (mesa area is indicated in each case).

To investigate the effect of size and aspect ratio of the patterned mesas on the elastic relaxation, we analyzed the AR-PFM domain maps and NND maps of the mesas with respect to domain orientation, as shown in Figs 3(a)–(e) and S6. The length of the mesas, parallel to the [100] direction, increased from 0.5 μm to 1.0 μm for fixed width of 0.5 μm (Fig. 3(a)–(c)), and the width of the mesas, parallel to [010] direction, was varied from 0.5 μm to 1.0 μm for fixed length of 1.0 μm (Fig. 3(c)–(e)).

The mesas with high aspect ratio – sizes 0.75 × 0.5 μm^2^ and 1.0 × 0.5 μm^2^ (Fig. 3(b),(c)) have the highest percentage of domains with a polarization variant angle of 225° (relative to the [100] direction). In addition, more than 75% of the domain polarization variants of the two patterned mesa structures have angles of 180–360° (the percentage of each domain variant is shown in Fig. 3(f)–(j) for the different mesas). The effect of the high aspect ratio is also found in the domain shape: Fig. S7 shows the aspect ratios of the domains for the mesas and unpatterned film. As can be seen, for the high aspect ratio mesas, the domain aspect ratio is also higher, with the domain shape preferentially elongated parallel to the long edges of the mesas. The observed results could also be explained by the depolarizing field created by imperfect charge screening of the patterned mesas^29,30^. The depolarizing field of the mesas with high aspect ratio is aligned in one direction due to the anisotropy of length and width, affecting the domain polarization variants and domain configuration.

**Figure 4:**
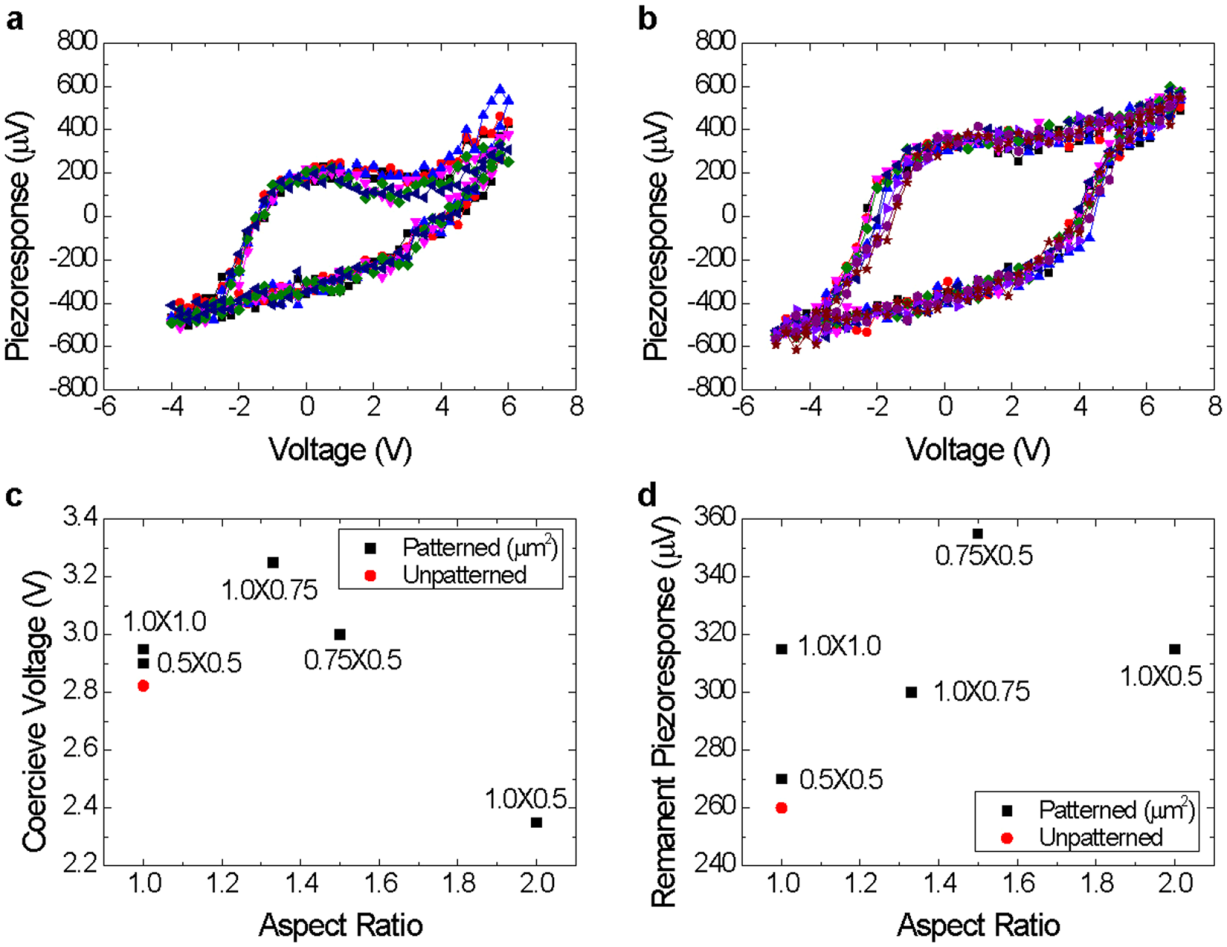
Piezoresponse loops for (**a**) unpatterned BFO film and (**b**) 0.75 µm × 0.5 µm patterned BFO mesa measured using local piezoelectric hysteresis loop measurement. (**c**) Coercive voltage and (**d**) remanent piezoresponse of the patterned BFO mesa structures and the unpatterned BFO film.

To correlate the ferroelectric domain configuration with the piezoelectric switching, we collected local piezoresponse hysteresis loops from the center of the patterned BFO mesas. Figures 4(a)–(b) and S8 show local piezoresponse loops for the BFO mesas and the unpatterned BFO film and their coercive voltage and remanent piezoresponse are shown in Fig. 4(c)–(d). The piezoresponse loops from the BFO mesas had a larger piezoresponse than that of the unpatterned BFO while their coercive voltages were similar, which implies that the release of constraint from the surrounding material possibly allowed for a higher piezoelectric strain to develop, which has been also observed for Pb(Zr,Ti)O_3_ mesas as well^5,31^.

In Fig. S9, the PFM amplitude appears to be linear with respect to the applied voltage and does not saturate. Such a linear relationship can be explained by the partial contribution of electrochemical strain mainly induced by the oxygen vacancy motion^32^. This unsaturated linear relationship became stronger in the unpatterned BFO film and the 1.0 × 1.0 µm^2^ patterned mesa, which also indicates that the release of constraint from the surroundings reduces defects such as oxygen vacancy. This phenomenon is well explained by other report, namely that epitaxial strain drives oxygen vacancies in BFO to diffuse from the relaxed surface to the strained interface, and that the STO substrate strongly absorbs oxygen vacancies from the BFO film^33^.

The local piezoresponse hysteresis loops of the BFO mesa structures showed different characteristics depending on the length and the width of the patterned mesas. The 1.0 × 0.5 µm^2^ patterned BFO mesa had the lowest coercive voltage and the 0.75 × 0.5 µm^2^ patterned BFO mesa had the largest remanent piezoresponse. Low coercive voltage or high remanent piezoresponse indicates easy polarization switching and/or enhanced piezoelectricity. As a result, it can be concluded that patterning of the BFO film with a wide mesa results in a simpler domain configuration, which leads to better piezoelectric characteristics.

## Conclusions

In summary, we have studied the ferroelectric domains of patterned (001) BFO films epitaxially grown on SRO/STO substrate using AR-PFM. We imaged the in-plane ferroelectric domains with angular increment of 30° and determined the degree of charging at the domain boundaries. We observed meta-stable polarization variants which the angle is not restricted to the <111> directions, which decrease the degree of charging at the domain boundaries. We compared the domain complexity of patterned and unpatterned BFO using the NND, and found out that patterned BFO had simpler domain structures due to the removal of the film surrounding the mesa structure of BFO. In addition, we found that the wider mesas showed simpler domain configuration and a better piezoresponse characteristics such as low coercive voltage or high remanent piezoresponse. Therefore, the patterning of thin films to mesa structures can be useful for nanoelectronic applications because of reducing the NND distribution.

## Methods

An epitaxial (35 nm) BFO/(70 nm) SrRuO_3_ (SRO) thin film heterostructure was grown on a (001)-oriented STO substrate and the BFO film was patterned into mesas ranging in size from 500 nm to 1.0 μm, using a combined electron-beam lithography and focused ion-beam (FIB) patterning procedure described elsewhere^12^. The details of how the ferroelectric domain maps were constructed are described in supplementary material. Nanoworld EFM-PtIr coated probes were used with an ac modulation voltage (1 V_rms_, 350 kHz for VPFM, 800 kHz for LPFM) for the PFM measurements in MFP-3D AFM of Asylum. Local piezoresponse loop was measured by PSIA XE-100 with lock-in amplifier (Standard Research Systems) using the same probes (1 V_rms_, 17 kHz, vertical mode).

## Electronic supplementary material

Supplementary Information
