# Supplementary material for: Ferroelectric Domain Studies of Patterned (001) BiFeO3 by Angle-Resolved Piezoresponse Force Microscopy
Source: Sci Rep. 2018 Jan 9;8:203. doi: 10.1038/s41598-017-18482-9 (PMC5760526; doi:10.1038/s41598-017-18482-9)
Supplement: Supplementary file 1 — Supplementary Information [file 41598_2017_18482_MOESM1_ESM.pdf]

Supplementary Information

Ferroelectric Domain Studies of Patterned (001) $BiFeO_3$ by Angle-Resolved Piezoresponse Force Microscopy

Bumsoo Kim[a,b], Frank P. Barrows[b,d], Yogesh Sharma[b,c], Ram S. Katiyar[c], Charudatta Phatak[b], Amanda K. Petford-Long[b,e], Seokwoo Jeon[a,*], and Seungbum Hong[a,b*]

[a] *Department of Materials Science and Engineering, KAIST, Daejeon 305-701, Korea.*
[b] *Materials Science Division, Argonne National Laboratory, Lemont, IL 60439, USA.*
[c] *Department of Physics and Institute for Functional Nanomaterials, University of Puerto Rico, San Juan, PR-00936-8377, USA.*
[d] *Applied Physics Program, Northwestern University, Evanston, IL 60208, USA*
[e] *Materials Science and Engineering Department, Northwestern University, Evanston, IL 60208, USA*

*Corresponding authors: jeon39@kaist.ac.kr and seungbum@kaist.ac.kr

Conventional and AR-PFM ferroelectric domain map

Various studies have reported that BFO polarization vectors can adopt one of eight thermodynamically-stable variants along the <111> crystallographic directions. Since the BFO film studied here only has a downward polarization vector component as shown in Fig. S1(a), the ferroelectric domain map can be constructed by combining the LPFM data with the cantilever direction aligned along [100] and [010]. Figures S2(a) and S2(d) show LPFM phase images of the unpatterned BFO thin film obtained with the cantilever direction aligned along [100] and [010] respectively (indicated by small white arrow). With respect to the cantilever direction, the black area represents ferroelectric domains that have polarization component to the right and the grey areas represents domains with polarization vector components to the left. Figure S2(g) shows a conventional ferroelectric domain map based on Fig. S2(a) and S2(d) contains up to four in-plane polarization variants.

However, as we increase the angular resolution from 90° to 30°, we can detect at least twelve polarization variants, including eight meta-stable polarization variants that do not lie along the <111> directions. If the in-plane piezoresponse is modulated by neighboring domains with different polarization directions, even if the polarization is restricted only to well-defined directions, the piezoresponse should change gradually. However, there are some domain boundaries at which the difference in polarization direction between the two domains is greater than 30°. In addition, the total percentage area of <111> polarization variants is less than 50% while the total percentage area of the non-<111> polarization variants is more than 50%. We believe that the total percentage area of non-<111> polarization variants is too large to be negligible or to be an error. Therefore, we believe that polarization directions are not restricted to the <111> directions.

Figures S2(a)–S2(f) show LPFM phase images of the BFO thin film obtained after rotating the sample by increments of 30°. Figure S2(i) shows an AR-PFM domain map based

on Fig. S2(a)–S2(f). Comparing Fig. S2(i) and Fig. S2(g), the AR-PFM domain map reveals more polarization variants than can be seen in the corresponding conventional ferroelectric domain map and therefore gives a more accurate view of the domain orientations. We have found through this that the large skeleton of the ferroelectric domain configurations is based on four in-plane <111> directions, but the meta-stable polarization variants also exist in significant proportions.

A similar problem is illustrated with the enlarged conventional domain map shown in Fig. S2(h): the image would suggest that some of the domain walls are charged and others are not. However, the AR-PFM domain map (Fig. S2(j)) shows a more accurate representation of the domain walls, indicating the way in which the metastable variants lead to lower charge distribution along the walls (see accompanying explanation in Fig. S3).

Constructing AR-PFM domain maps

To construct AR-PFM domain map, LPFM phase signals with topography images were measured after each rotation around the film surface normal, from 0 ° to 150 ° with an interval of 30 ° between each domain image. The direction of the in-plane piezoresponse is not always along to the direction of the polarization because the in-plane piezoresponse is different from the original piezoelectric coefficients of d_{15}, d_{22}, d_{31} and d_{33} in the rhombohedral crystal symmetry. However, in this research, we believe that in the region where the LPFM phase changes when we rotate the cantilever, the polarization direction does lie between the adjacent cantilever axes (either $(\theta_a+\theta_b)/2$ or $(\theta_a+\theta_b)/2+180°$) due to the following reasons:

First, for spontaneous polarization aligned parallel to the surface, the shearing due to d_{15} provides the dominant contribution to the lateral tip movement in the pseudocubic case. In our epitaxially-grown BFO film on a SRO/STO substrate, the crystal symmetry is closer to cubic as reported in S. Hong *et al.*, J. Appl. Phys. **105**, 061619 (2009). In addition, d_{33} can

affect the lateral piezoresponse at the domain boundary between upward and downward polarization domains. However, since the BFO film studied here only has a downward polarization vector component as shown in Fig. S1(a), the influence of d_{33} on the in-plane piezoresponse is negligible. Finally, when we rotate and measure LPFM in the resolution of 30°, a change of LPFM phase between two adjacent scanning angles indicates that a change in the polarization direction in the resolution of 30° even if d_{zx} is affected by not only d_{15} but also other tensors, d_{31}, d_{22}, and d_{33}.

Each set of LPFM phase images was rotated and aligned based on the topography images. All sets of registered LPFM phase images were bin to three regions as shown in Fig. S10. Black and white regions represent left and right vectors based on the cantilever direction and grey region represents unknown vector because the phase value is unclear. Preprocessed phase images were merged into an incomplete ferroelectric domain map. We identified each in-plane polarization vector by finding the area where the phase contrast changed by 180° after two adjacent rotations of the sample, and denoted the in-plane component of the polarization vector for that area as lying between the scan directions before and after the phase reversal.

The incomplete ferroelectric domain map has undefined black and white area as well as defined 12 colored ferroelectric vectors. Black region has no vector information and white region has more than two vectors. To remove these errors and complete the ferroelectric domain map, we assigned the value to each pixel considering nested vectors or the nearest pixels iteratively.

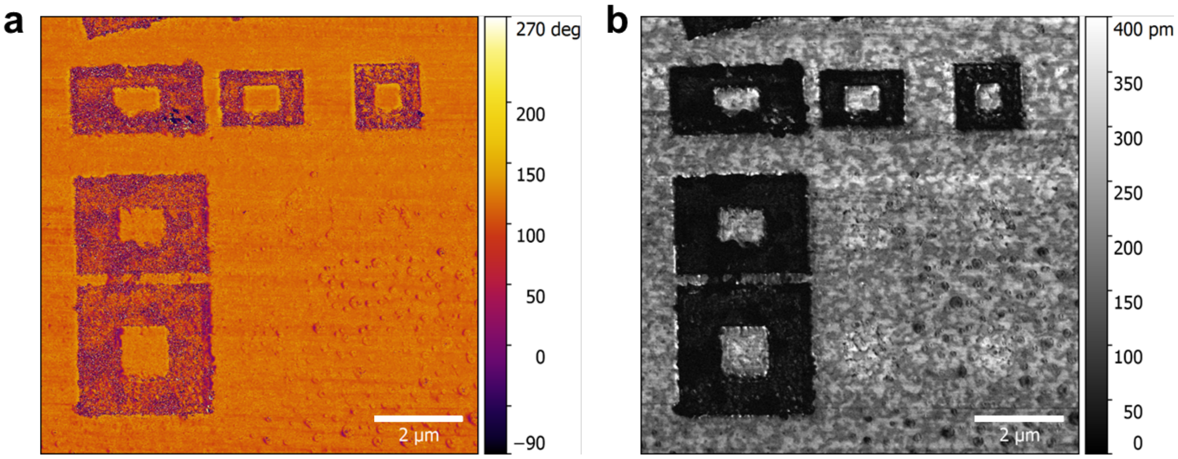

Figure S1 (a) VPFM phase and (b) amplitude image of the BFO film. The phase values of etched area show a noise signal and the amplitude values of etched area converged to 0 because SRO/STO substrate have no piezoresponse characteristics.

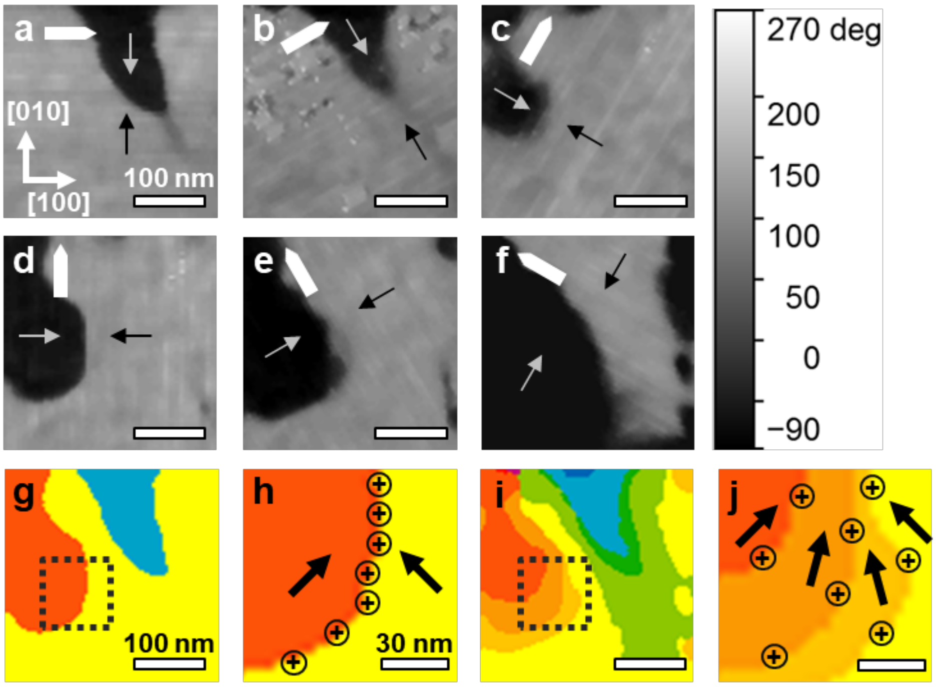

Figure S2 (a)-(f) LPFM phase images of a BFO thin film obtained with incremental rotation of the sample by 30 °. The images were rotated and fitted based on the associated topography images. Based on the cantilever direction, the grey areas represents ferroelectric domains that have polarization vectors pointing to the left and the black areas have ferroelectric vectors pointing to the right. (g),(h) Conventional ferroelectric domain maps constructed using only the 0 and 90 ° images (i.e. only (a) and (d)) based on the conventional model. (i),(j) AR-PFM domain maps constructed using the 0, 30, 60, 90, 120, and 150 ° images.

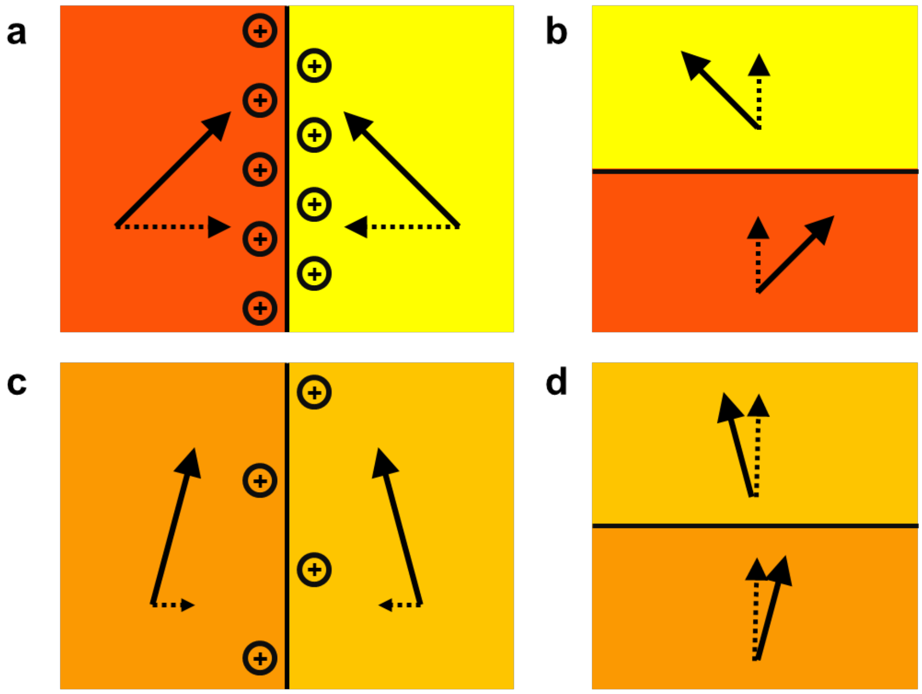

Figure S3 (a),(c) The domain boundary is charged due to a head-to-head polarization along the boundary normal direction. The accumulated charge decreases as the angle between ferroelectric vector and the domain boundary decreases. (b),(d) The domain boundary is uncharged due to a head-to-tail polarization along the boundary normal direction.

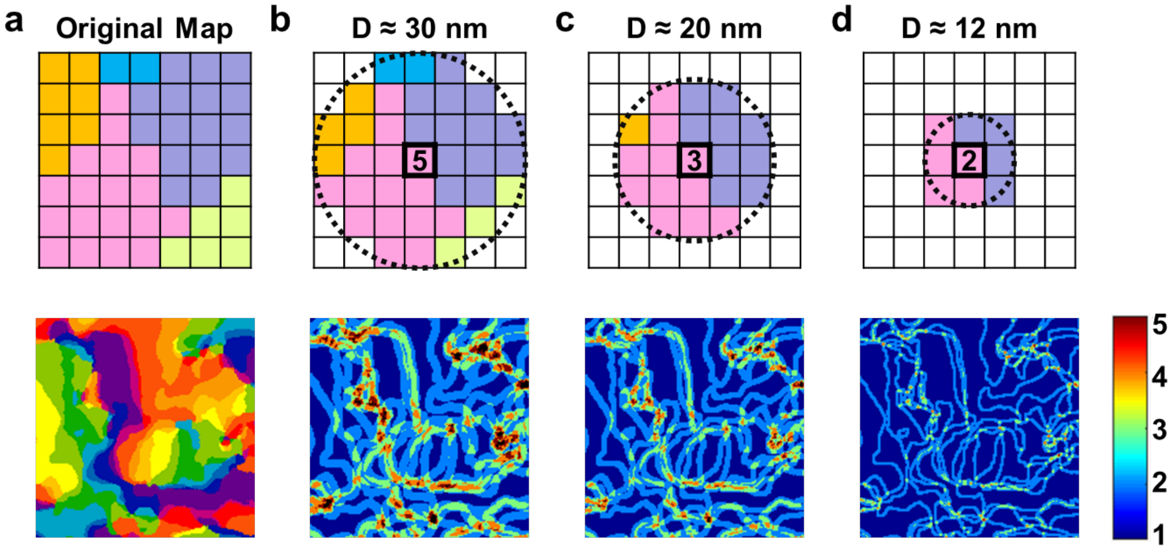

Figure S4 Explanation of NND analysis (a) region of ferroelectric domain, (b)–(d) NND analysis for different diameters D. The lower row shows the entire domain map together with the NND analysis for different values of D. Color scale indicates the number of neighboring domains at each point.

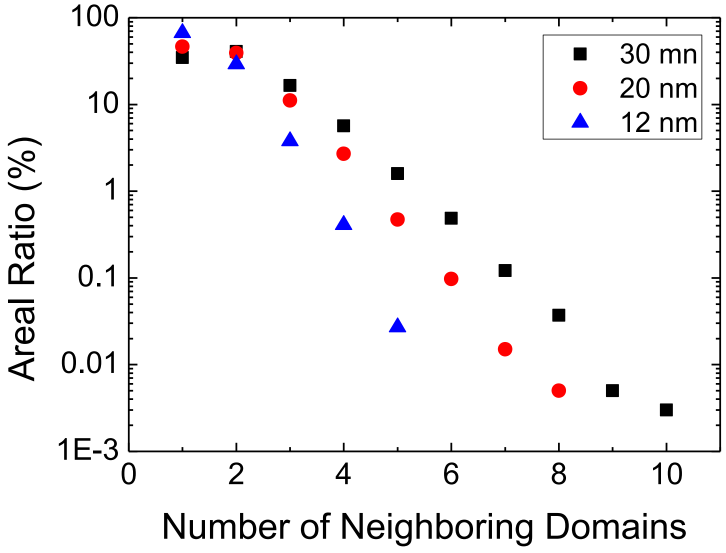

Figure S5 Area distribution of NND for different values of *D* plotted on a log-linear scale.

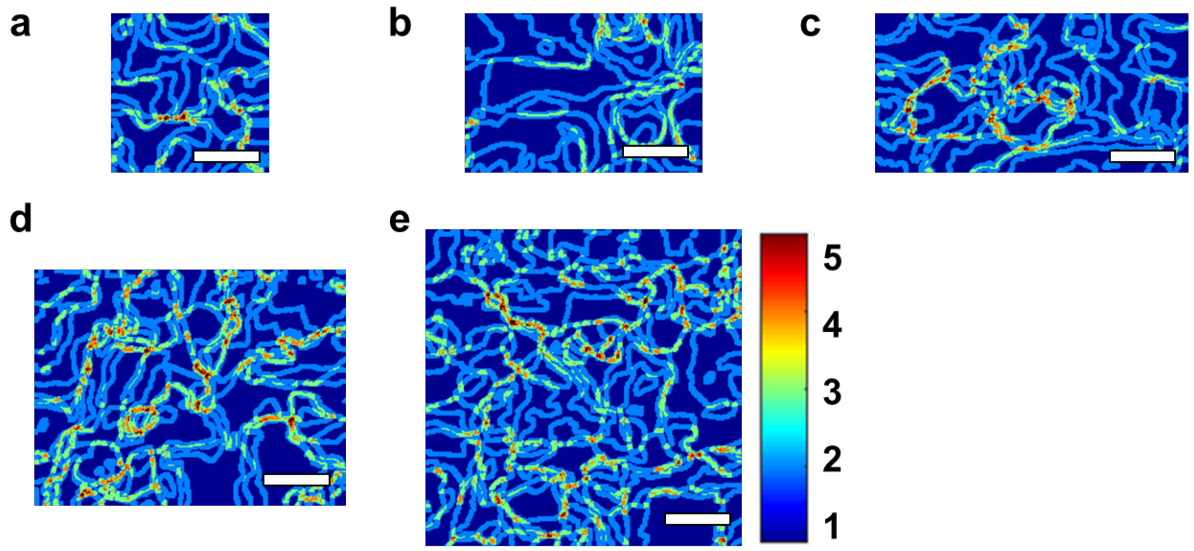

Figure S6 NND maps for patterned BFO mesas of size (a) 0.5 μm × 0.5 μm, (b) 0.75 μm × 0.5 μm, (c) 1.0 μm × 0.5 μm, (d) 1.0 μm × 0.75 μm, and (e) 1.0 μm × 1.0 μm. Color bar corresponds to the number of neighboring domains (NND) at each pixel position

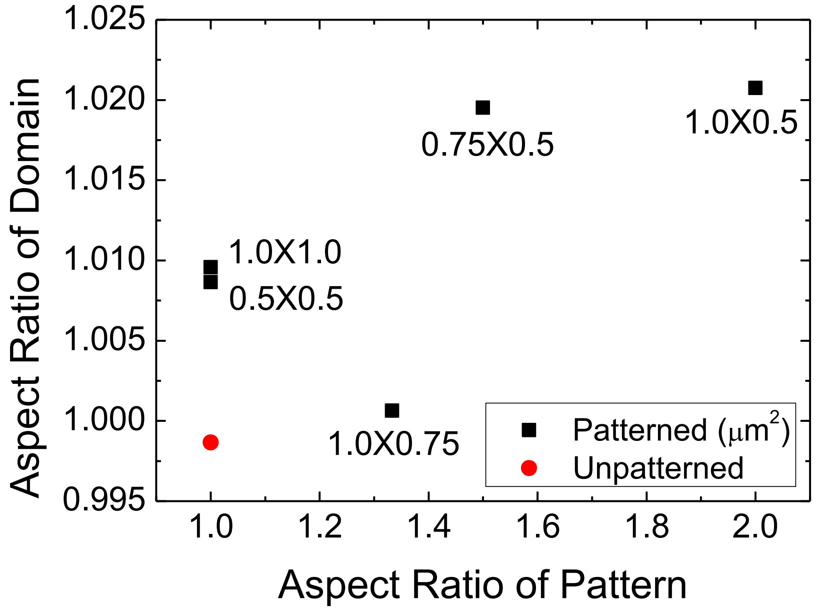

Figure S7 Plot of aspect ratio of domains versus aspect ratio of mesas. Aspect ratio of the domain is ∑(horizontal component of domain boundary)/∑(vertical component of domain boundary). High domain aspect ratio means that the domain is wide.

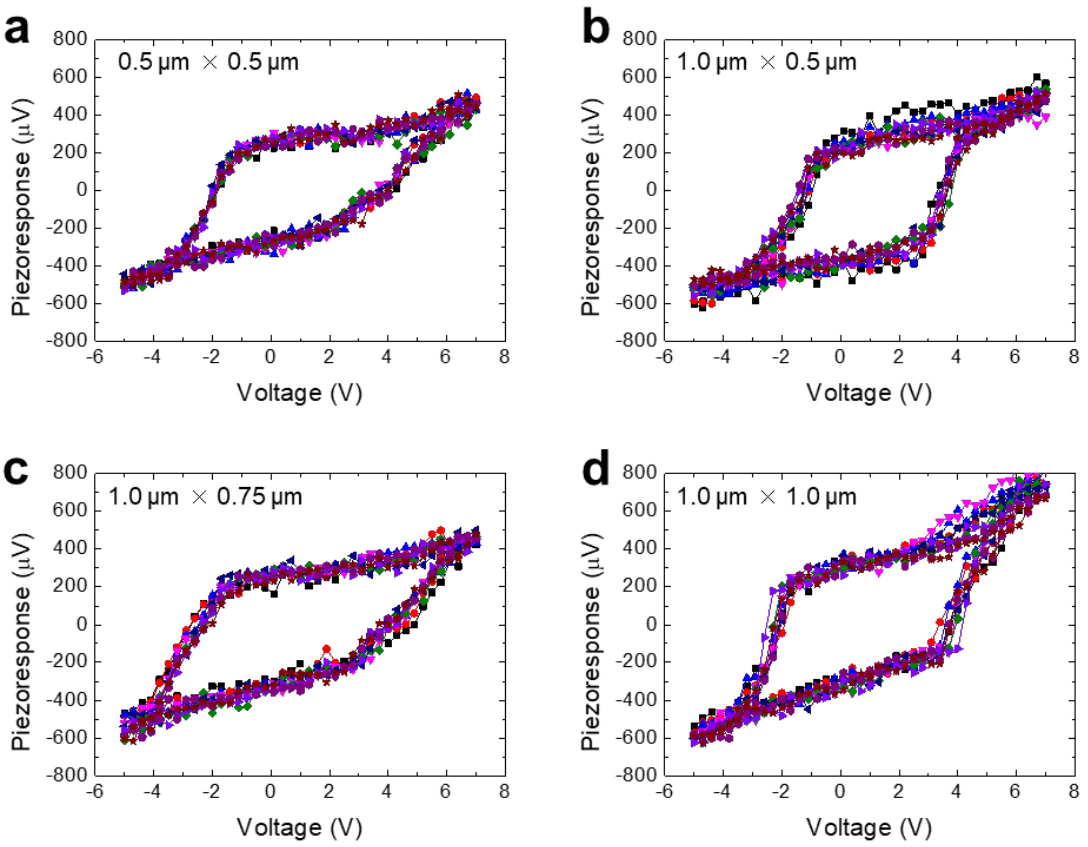

Figure S8 Piezoresponse loops for (a) 0.5 μm × 0.5 μm, (b) 1.0 μm × 0.5 μm, (c) 1.0 μm × 0.75 μm, and (d) 1.0 μm × 1.0 μm BFO mesa structures measured using a local piezoelectric hysteresis loop measurement.

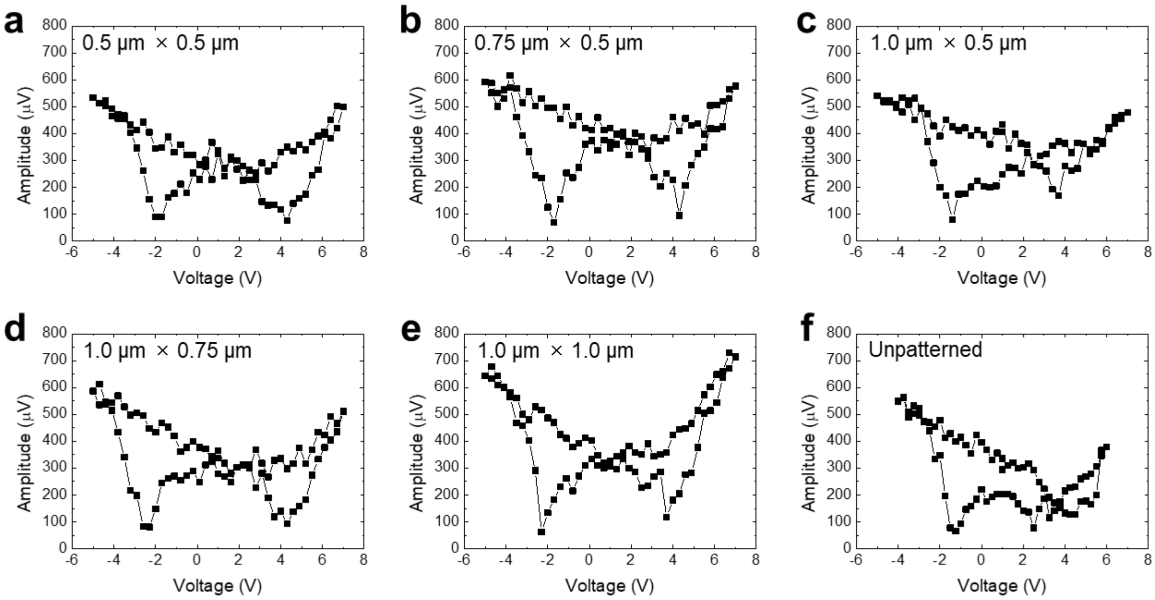

Figure S9 Amplitude curves for (a) 0.5 µm × 0.5 µm, (b) 0.75 µm × 0.5 µm, (c) 1.0 µm × 0.5 µm, (d) 1.0 µm × 0.75 µm, and (e) 1.0 µm × 1.0 µm BFO mesa structure and (f) unpatterned BFO film measured using VPFM.

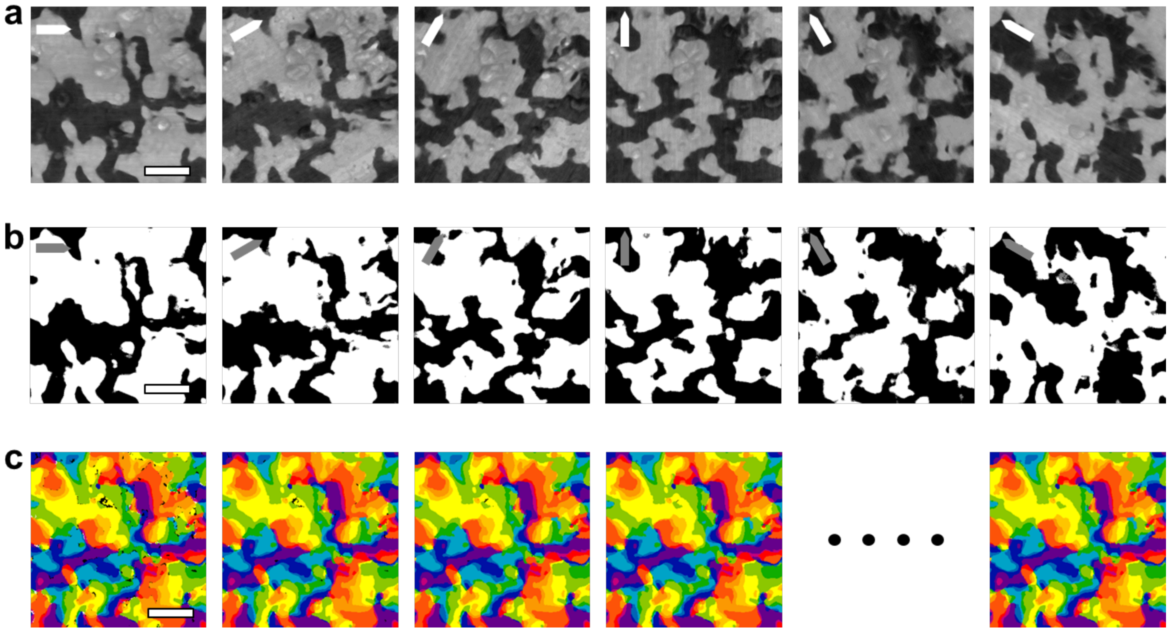

Figure S10 (a) LPFM phase images of unpatterned BFO film obtained after sample rotating by 30 ° were registered using topography images. (b) Depending on the value of the LPFM phase signals, the right vectors of the cantilever direction is black, the left vectors of the cantilever direction is white, and the unclear region is grey. (c) The undefined pixels of ferroelectric domain map are iteratively removed by assigning the value to each undefined pixel considering the nearest pixels.
